# The role of periodontal treatment on the reduction of hemoglobinA1c, comparing with existing medication therapy: a systematic review and meta-analysis

**DOI:** 10.3389/fcdhc.2025.1541145

**Published:** 2025-02-25

**Authors:** Yojiro Umezaki, Akiko Yamashita, Fusanori Nishimura, Toru Naito

**Affiliations:** ^1^ Section of Geriatric Dentistry, Department of General Dentistry, Fukuoka Dental College, Fukuoka, Japan; ^2^ Department of Periodontology, Division of Oral Rehabilitation, Faculty of Dental Science, Kyushu University, Fukuoka, Japan

**Keywords:** periodontology, diabetes mellitus, periodontal initial treatment, hemoglobinA1c, C-reactive protein

## Abstract

**Background:**

Diabetes mellitus (DM) is linked to complications such as retinopathy, nephropathy, neuropathy, and cardiovascular disease, impacting patient quality of life and increasing healthcare costs. Periodontal disease, more prevalent in diabetic patients, is associated with worsened glycemic control and systemic inflammation, suggesting a possible bidirectional relationship. While some studies indicate periodontal treatment may improve glycemic control and reduce inflammation, overall evidence is inconsistent. It remains unclear if periodontal therapy reliably enhances diabetes outcomes or if certain patient subgroups benefit more than others.

**Objective:**

To systematically review randomized controlled trials (RCTs) evaluating the effects of periodontal therapy on glycemic control (HbA1c) and systemic inflammation (CRP) in type 1 and type 2 diabetes patients.

**Methods:**

Following PRISMA guidelines, a comprehensive PubMed search identified RCTs comparing HbA1c and CRP outcomes in diabetic patients with periodontal therapy versus controls. Inclusion criteria required at least three to six months of follow-up. Meta-analyses using a random effects model were conducted for HbA1c and CRP changes.

**Results:**

Eleven studies met inclusion criteria. Meta-analyses showed significant reductions in HbA1c at three months (-0.64; CI95%=-0.96 to -0.32; I2 = 73%) and six months (-0.33; CI95%=-0.65 to -0.01; I2 = 12%). CRP also declined significantly, indicating an improvement in systemic inflammation.

**Conclusion:**

Periodontal therapy appears to significantly reduce HbA1c and CRP levels over short-term periods in diabetic patients, suggesting potential as a beneficial adjunct to diabetes management. These findings support incorporating periodontal care into diabetes treatment to reduce systemic inflammation and potentially lower healthcare costs. Future long-term, standardized RCTs are needed to confirm sustained effects and investigate responses in diverse patient populations.

## Introduction

Diabetes mellitus is known to cause complications such as retinopathy, nephropathy, and neuropathy, and is also known to be involved in the onset and progression of arteriosclerotic diseases such as ischemic heart disease and stroke. These complications not only significantly reduce the quality of life of patients, but also impose a heavy medical and economic burden on society, for which countermeasures are required.

In the oral cavity, periodontal disease is also recognized as a complication because of its high frequency in diabetic patients, and many studies have been conducted on the relationship between diabetes and periodontal disease. The “Guidelines for Periodontal Treatment of Diabetic Patients, Revised Third Edition 2023,” (1) published by the Japanese Society of Periodontology, states that diabetic patients, whether type 1 or type 2, have a significantly higher incidence of periodontal disease than non-diabetic patients. A meta-analysis of cohort studies on the bidirectional association between periodontal disease and diabetes reported a 24% increased incidence of periodontal disease in diabetic patients and a 26% increased relative risk of developing diabetes in patients with periodontitis (2). A recent review by the European Federation of Periodontology (EFP) and the American Academy of Periodontology (AAP) (3) also found that those with periodontitis were more likely to develop type 2 diabetes (hazard ratio 1.19-1.33) than those without the disease. In other words, type 1 and type 2 diabetics are significantly more likely to develop periodontal disease than non-diabetics.

Diabetes has also been shown to be a risk factor involved in the progression of periodontal disease. For example, cross-sectional studies examining the relationship between duration of diabetes and periodontal disease have shown that attachment loss is greater and periodontal disease worsens when duration of type 1 and type 2 diabetes exceeds 5 years (4, 5).

In a study of 7042 U.S. National Nutrition Examination Survey (NHANES III) (6) physical examiners with periodontal examination findings and fasting blood glucose data, the HbA1c of patients with periodontal disease was 5.9, compared with 5.6 in patients without periodontal disease and 5.6 in patients with diabetes mellitus. In a study of the relationship between glycemic control status and periodontal disease, the HbA1c of patients with periodontal disease was 5.9, compared with 5.6 in patients without periodontal disease. In the case of diabetic patients, the HbA1c values increased to 7.4 and 7.0 in periodontal patients and non-diabetic patients, respectively, and the condition of periodontal tissues deteriorated in patients with HbA1c≥8.0 in terms of glycemic control status. In addition, previous reports also showed that the glycemic control status of periodontal tissues deteriorated in patients with HbA1c≥8.0. In an earlier report (7), the risk of periodontitis was 2.9 times higher in type 2 diabetics with extremely poor glycemic control (HbA1c > 9%) than in non-diabetics, but there was no statistically significant difference in the risk in patients with HbA1c ≤ 9%, although there was a trend toward more advanced periodontitis than in non-diabetics. Furthermore, it has been shown that the risk of alveolar bone resorption is higher in very poor type 2 diabetics with HbA1c ≥ 9% (8). With regard to glycemic control in type 1 diabetes, it has been reported that alveolar bone resorption is more common in poorly glycemic controlled diabetics compared to those with good glycemic control (9).

On the other hand, the relationship between improvement of diabetes and periodontal treatment is controversial. Grossi et al. (10) reported that periodontal therapy improved diabetes in a randomized control trial, and many other reports have been published, but some reports (11, 12) showed that the effect of periodontal therapy on diabetes is negative. In this study, we conducted a systematic review of previous reports on the improvement of diabetes mellitus and periodontal treatment.

## Subjects, materials, and methods

This systematic review adhered to the Preferred Reporting Items for Systematic Reviews and Meta-analysis (PRISMA) guidelines (13). The PICO question we investigated in this review was formed according to the rules of PRISMA.

“Do the patients with Type 2 Diabetes Mellitus (P) with periodontal therapy (I), compared to without treatment (C), improve HbA1c (O1) or CRP (O2).”

### Inclusion and exclusion criteria

Studies were included, if they (1) were randomized controlled studies (2); included patients over the age of 20 (3); observed at least 3 or 6 months. Studies were excluded if they (1) did not report on any of the predefined outcomes (2); were animal studies (3); were inadequate article types, such as notes, reviews, letters or conference abstracts (4); had high risk of bias.

### Search strategy

For this review, a PubMed search was first conducted, and in filtering, the literature was narrowed down using Clinical Trial, considering the nature of this clinical question. The complete search key used was the following: (“Periodontitis” [MeSH Terms] OR “Periodontal Diseases” [MeSH Terms]) AND (“Diabetes Mellitus” [MeSH Terms]) AND (“Glycemic Control” [All Fields] OR “Hemoglobin A1c [MeSH Terms]) AND (“Periodontal Therapy” [All Fields]) AND (“Intervention” [All Fields]), filters: (“Humans”, “English” and “Clinical trial”). The databases were screened on Sep 1, 2021.

For references that were deemed appropriate for inclusion in the PubMed search, we further reviewed similar articles in PubMed, examined the titles of references cited in the article, and conducted additional literature searches. The final pool of included studies was decided upon completing the full-text selection procedure.

### Data extraction and quality assessment

Two authors independently extracted the required data. The following information was collected: first, the year of publication; second, the authors’ names; and third, the study title. Data on changes in HbA1c, CRP, PCR, BoP, and PPD were compiled. Some studies also reported hs-CRP results, which were extracted as well. The results and conclusions of each study were summarized to facilitate easier comparison and make the findings more readily accessible. Quality assessment was conducted in accordance with Cochrane guidelines (14). Any disagreements between the two authors were resolved through discussion with the corresponding author to reach a consensus.

### Statistical analysis

We assessed changes in HbA1c levels and CRP from baseline to post-treatment as primary outcomes, reflecting treatment-related improvement. Weighted mean difference and corresponding 95% confidence intervals (CI) were calculated by the forest plot. A random effect model was employed, and studies were weighed by a classical inverse variance method (15). Heterogeneity was examined using I2 value. Review Manager (Revman) version 5.4 for Windows from Cochrane collaboration was used to perform all analyses. Statistical significance has been defined as a P value < 0.05.

## Results

### Search results

**Figure 1:**
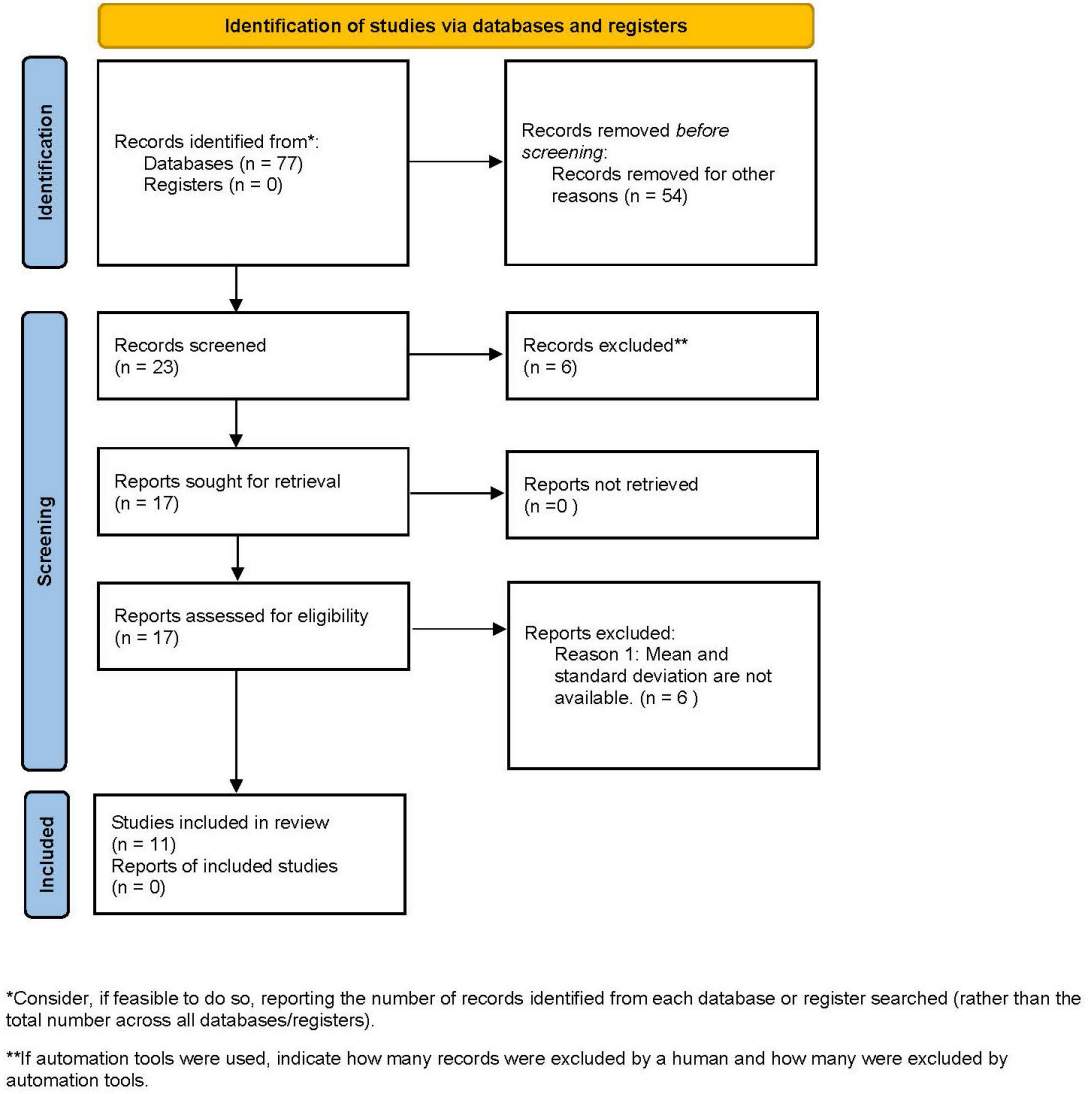
Flow chart of the literature search and screening process.

From the systematic search 77 articles were retrieved and assessed by title and abstract selection. Conducting the full text selection, 11 RCTs fulfilled the eligibility criteria and were processed for data extraction. The databases were screened on Sep 1, 2021. No additional eligible studies were found at the manual hand searches of the reference lists. The detailed selection procedure can be found in **Figure 1**. The characteristics of the 11 RCTs were shown in **Table 1**. Among them, 10, 6, 2 and 2 studies reported 3-months outcomes for HbA1c, 6-months outcomes for HbA1c, 3-months outcomes for CRP and 6-months outcomes for CRP, respectively. There were 3 studies (19, 24, 25) that compared three groups between two kinds of treatment and controls. Two (19, 25) of these studies manually combined the treatment groups and considered 2-group comparisons. In the remaining study (24), one of the subgroups was not considered clinically meaningful, so we excluded the corresponding group and dealt with as a two-group comparison.

**Table 1:** Study characteristics (n=11).

Authors year (country)	Study design	Number of participants	Mean (SD) or range of years of age	Gender (M/F)	Number of smokers	Dropout	DM definition	periodontitis definitions	Intervention	Follow-up visits	Outcome for glycemic parameters	Outcome for periodontal measurements	Systemic inflammation
Kaur PK, et al., 2015 (16) (India)	RCT	100	52.38 (5.94)	48/52	0	9	Diagnosed T2DM for ≥ 1 year	Moderate periodontitis was defined as two or more interproximal sites, not on the same tooth, with CAL ≥4 mm or PPD ≥5 mm. Severe periodontitis was defined as two or more interproximal sites, not on the same tooth, with CAL ≥6 mm, and one or more interproximal sites with a PPD ≥5 mm. ≥ 12 teeth	Treatment group: SRP No-treatment group: No intervention	3 and 6 months ITT analysis	HbA1c: Significant difference between groups at 3 and 6 months (p < 0.05)	PPD, CAL, GBI, PI, BOP%, PESA and PISA were improved significantly in treatment group after 3 and 6 months than those in the no-treatment group (p < 0.05).	No information
Khader, et al., 2010 (17) (Jordan)	RCT	58	56.38 (7.38)	22/28	22	8	HbA1c level ≥7%	Loss of 75% supporting bone, pocket depth > 8mm, class III furcation involvements using Glickman index, class III mobility using Miller index, poor crown-to-root ratio, root proximity with minimal interproximal bone, history of repeated periodontal abscesses, and usefulness of prosthetics and restorative ≥ 8 teeth	Treatment group: Fullmouth tooth extraction Control group: OHI	3 and 6 months PPS analysis	HbA1c: Significant reduction at 3 and 6 month follow-up in treatment group (p < 0.05). No significant change at 3 and 6 months in control group (p > 0.05).	No information	No information
Mizuno, et al., 2017 (18) (Japan)	RCT	37	61.94 (10.53)	28/9	7	9	Diagnosed T2DM for ≥ 2 months	≥ 2 interproximal sites with CAL ≥ 3 mm and ≥ 2 interproximal sites with PPD ≥ 4 mm (not on the same tooth) or one site with PPD ≥ 5 mm	Treatment group: SRP + OHI Control group: OHI only	3 and 6 months	HbA1c: No significant differences at 3 and 6 months follow-up between Treatment group and control (p > 0.05).	Changes in the PPD and CAL at 3 and 6 months significantly differed between the periodontal treatment and control groups. Changes in the percentage of CAL≥4mm and BOP significantly differed between the periodontal treatment and control groups at 6 months.	Systemic oxidative stress balance significantly improved in the treatment group compared to the control group at 3 months (p < 0.05).
Qureshi, et al., 2021 (19) (Pakistan)	RCT	150	52.26 (7.55)	82/68	8	76	Diagnosed T2DM for ≥ 1 year, HbA1c level ≥6.5% and < 14%	≥ 2 interproximal sites having ≥ 5 mm PPD or ≥ 4 mm of CAL ≥ 16 teeth	Group1: SRP with MET and OHI Group2: SRP + OHI Control: OHI only	3 and 6 months	HbA1c: Significant reduction at 3 and 6 month follow-up in G1 and G2 (p < 0.05). Significant increase at 3 and 6 months follow-up in Control (p < 0.05).	BOP%, PPD, CAL at 1 and 3 months in G1 and G2 significantly decreased (p < 0.05).	No information
Tsobgny-Tsague, et al., 2018 (20) (Cameroon)	RCT	34	51.45 (8.85)	13/17	0	4	Poorly controlled T2DM patients	Moderate to severe chronic periodontitis according to the 2012 CDC-AAP classification ≥ 11 teeth	Treatment group: SRP + OHI Control group: OHI only	3 months PPS analysis	HbA1c: Significant reduction at 3 months in treatment group (p < 0.05). No significant change at 3 months in control group (p > 0.05).	PI, GBI, PPD and CAL in treatment group significantly decreased (p < 0.05). Plaque index in control group significantly decreased (p < 0.05).	No information
Wei-Lian Sun, et al., 2011 (21)(China)	RCT	190	54.70 (11.01)	67/90	0	33	Diagnosed T2DM for ≥ 1 year, HbA1c level > 7.5% and < 9.5%	More than 30% teeth with PPD > 5 mm and CAL > 4 mm, or over 60% teeth with PPD >4 mm and CAL >3 mm≥ 20 teeth	T2DM-T group: OHI + SRP + periodontal flap surgery when indicated + extraction of hopeless teeth + restore of balanced occlusion + Antibiotics prescriptionT2DM-NT group: No intervention	3 monthsPPS analysis	HbA1c: Significant difference between groups at 3 months (p < 0.05)	PPD, CAL, GBI, and PI, were improved significantly in T2DM-T group after 3 months than those in the T2DM-NT group (p < 0.05).	hs CRP, TNF-α, IL-6,significantly decreased in T2DM-T group than T2DM-NT group at 3 months (p < 0.05).
D’Aiuto F, et al., 2018 (22) (UK)	RCT	264	56.86 (9.85)	165/99	119	20	Diagnosed T2DM for ≥ 6 months	≥20 periodontal pockets with probing pocket depths of >4 mm and marginal alveolar bone loss of >30% ≥ 15 teeth	IPT: whole mouth subgingival scaling, surgical periodontal therapy, and SPT every 3 months CPT: supra-gingival scaling and polishing	2, 6, 9 and 12 months ITT analysis	HbA1c: No significant difference between groups at 6 months (p > 0·05) and a significant difference at 12 months (p <0.05).	Full mouth dental plaque scores, bleeding scores, PPD and number of deep periodontal pockets were significantly better in the IPT group than CPT group at 2, 6, 12 months (p < 0.05).	CRP significantly decreased in IPT group than CPT group at 2, 6 and 12 months (p < 0.05).
Promsudthi, et al., 2005 (23) (Thailand)	RCT	52	61.36 (5.82)	19/33	0	0	Uncontrolled T2DM, HbA1c level > 7.5% and < 11.0%	At least 14 teeth with severe periodontitis as defined by at least eight sites with pocket depth ≥5 mm and clinical attachment level ≥5 mm.	Treatment group: OHI+ SRP+ DOXY Control group: No intervention	3 months	The reduction in the levels of FPG (p > 0.05) and HbA1c (p > 0.05) did not reach significance in treatment group.	PI, BOP, PPD,and attachment loss in treatment grope were significantly decreased(p < 0.05).	No information
Chen L, et al., 2012 (24) (China)	RCT	134	60.28 (9.90)	68/66	26	8	Diagnosed T2DM for ≥ 1 year	Moderate, and severe periodontitis, with a ≧1mm mean clinical attachment loss, ≧16 teeth.	Treatment group 1: SRP at baseline and additional subgingival debridement at the 3-month follow-up. Treatment group 2: Non-surgical periodontal treatment at the initial visit and only supragingival prophylaxis, with no intervention in deep periodontal pockets at 3 months. The control group: No intervention	1.5, 3, and 6 months	Although HbA1c declined significantly in treatment group 2 (p < 0.05), the intergroup difference for HbA1c, FPG, TNF-α, and lipid profiles was not statistically significant after therapy (p > 0.05).	In both treatment groups, PI and BOP decreased significantly at 1.5,3, and 6 months after the treatment. In the control group, PI and BOP declined significantly at 1.5 months compared to baseline values (p < 0.05). The percentage of sites with PD = 4 to 5mm and PD ≥6 mm declined significantly over time in both treatment groups (p < 0.05), whereas it did not change significantly in the control group throughout the study period (p > 0.05).	Both treatment groups had a significantly lower hsCRP level after periodontal therapy (p <0.05).
Das AC, et al., 2019 (25) (India)	RCT	51	45.92(7.88)	29/22	0	0	Controlled T2DM patients	Moderate to severe periodontitis(30% or more of the teeth have ≥4 mm CAL)	Group I: SRP Group II: SRP+DOXY GroupIII(control): No intervention	3 months (Day 0 to 90)	FPG, PPG, and HbA1c level were reduced in groups I and II compared to group III; however, only HbA1c values were found significantly reduced (p < 0.05) at day 90.	The mean difference between baseline and day 90 for all periodontal parameters (PI, GI, PPD, and CAL) were significantly higher (p < 0.05) in group I and II compared to control.	No information
El-Makaky, et al., 2020 (26) (Egypt)	RCT	88	52.59 (6.78)	38/50	0	0	Diagnosed T2DM for ≥ 5year, Uncontrolled T2DM HbA1c level > 7% and < 9%	Chronic periotontitis, CAL and PD ≥4 mm in more than 30% of the sites, the presence of 4 teeth as a minimum with at least one site with a CAL ≥3 mm and PPD ≥4 mm.	Test group: SRP, a combination of systemic antibiotics (MET and AMPC) + OHI Control group: No intervention	3 months ITT analysis	HbA1c: Significant reduction at 3 months in test group (p < 0.05).The control group, it was significantly increased from baseline to termination of trial.	PPD, CAL, and BOP were improved significantly in treatment group after 3 months than those in the no-treatment group (p <0.05).	No information

T2DM, Type 2 diabetes mellitus; SRP, Scaling root plaining; MET, metronidazole; OHI, oral hygiene instructions; PPD, Periodontal pocket depth; CAL, Clinical attachment loss; IPT, Intensive periodontal treatment; CPT, Control periodontal treatment; SPT, Supportive periodontal therapy; PI, Plaque index; GBI, Gingival bleeding index; PESA, Periodontal epithelial surface area; PISA, Periodontal inflammatory surface area; AMPC, Amoxicillin; DOXY, Doxycycline; FPG, Fasting plasma glucose; PPG,2-hour postprandial plasma glucose.

In studies with 3-months follow-up time for the change of HbA1c, the number of patients ranged from 30 (20) to 190 (21) with medically diagnosed type 2 DM. Mean age ranged from 45.9 (25) to 61.9 (18) years, and proportion of females varied from 24.3% (18) to 63.5% (23). Six studies did not include smokers, but other 4 studies included smokers ranging from 5.3% (19) to 37.9% (17) of the participants. In most of the studies, the participants did not receive any periodontal treatment for at least 3 months, whereas 2 studies (17, 19) not providing its information. As for studies with 6 months follow-up time for the change of HbA1c, sample size was from 37 (18) to 264 (22). The mean age was ranging from 52.3 (19) to 61.9 (18) years old. Four studies had equal proportions of male and female participants, but 2 studies had relatively low proportions of female participants, 24.3% (18) and 37.5% (22), respectively. Most of studies included smokers ranging from 5.3% (19) to 45.1% (22), but a study excluded smokers (16). Three studies (16, 18, 24) excluded the patients receiving periodontal treatment prior at least 6 months, while the other studies had no information on it.

On the other hand, 2 studies reported 3 months follow-up for CRP. The number of patients were ranging from 134 (24) to 190 (21). The mean ages were 60.3 (24) and 54.7 (21). Both studies had similar ratios of male to female. And 2studies showed the result of the change of CRP for 6 months follow up. The sample size was from 134 (24) to 264 (22). The mean ages were 60.3 (24) and 56.9 (22) years old. Female ratios were 49.3% (24) and 37.5% (22).

### Risk of bias assessment

**Figure 2:**
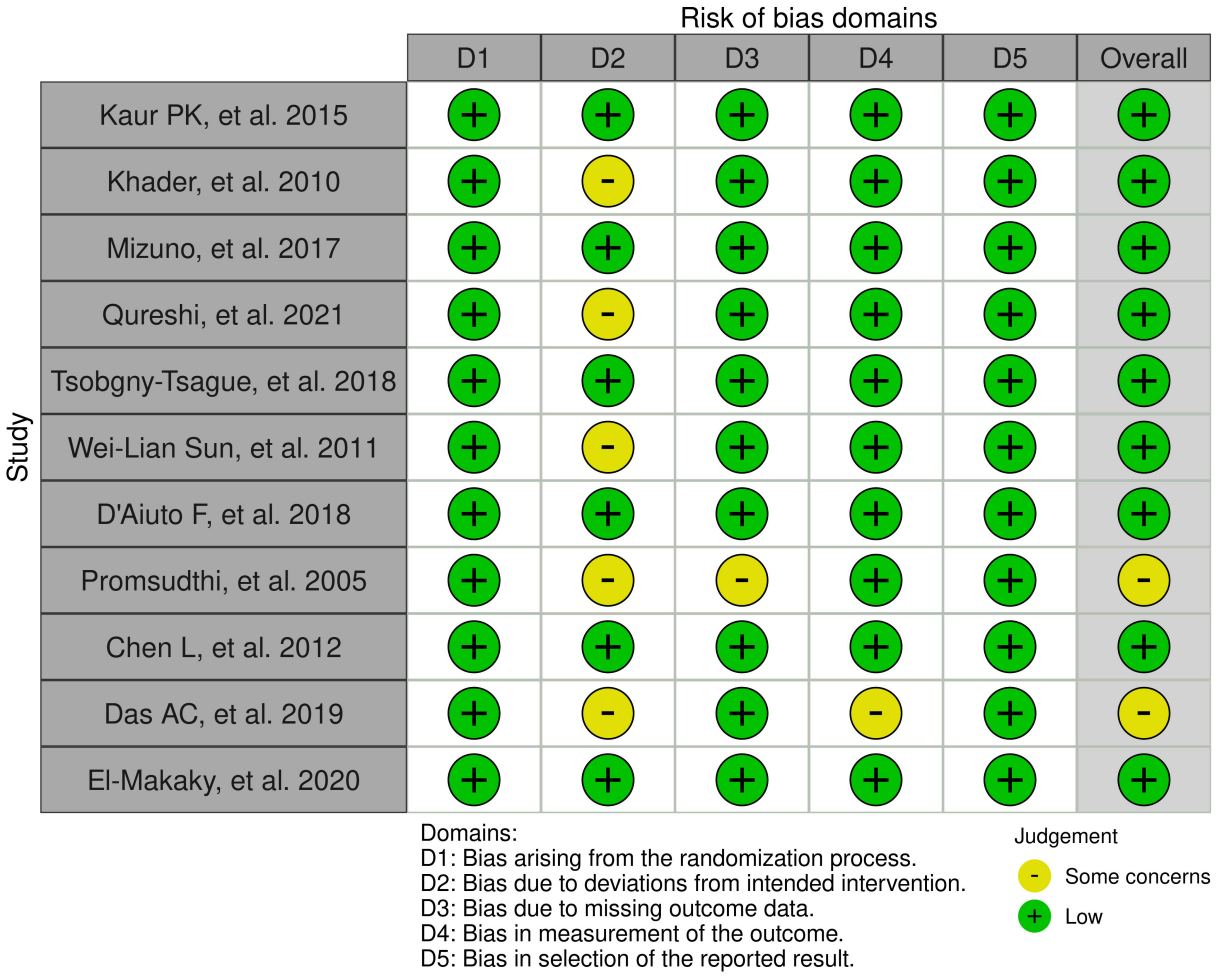
Summary of the risk of bias.

Eight studies were classified as having a low risk of bias, while the remaining two studies were categorized as having some concerns. No study was identified as having a high risk of bias (**Figure 2**). Most trials detailed their randomization process, often utilizing computer-generated random numbers, permuted block method, and opaque envelopes for allocation concealment. The primary sources of potential bias were related to the lack of blinding of participants or personnel and the loss of follow-up data.

### Results of the meta-analysis

**Figure 3:**
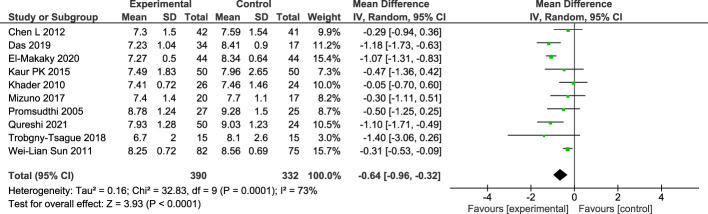
Forest plot for the change of HbA1c between baseline and 3-months follow-up.

There were 10 studies included in the meta-analysis of HbA1c changes in 3 months (16–21, 23–26). A total of 332 subjects in the control group and 390 subjects in the treatment group were analyzed for changes of HbA1c levels using random effect model. There was a statistically significant difference between patients in control group and treatment group with a result of -0.64 (CI95%=-0.96; -0.32). The between study heterogeneity was considered moderate; I2 = 73% (**Figure 3**).

**Figure 4:**
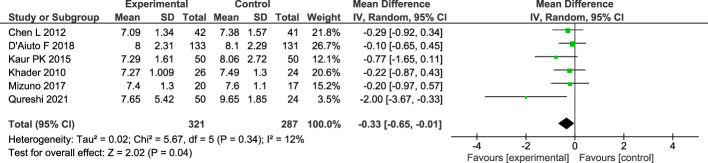
Forest plot for the change of HbA1c between baseline and 6-months follow-up.

As for 6 months changes in HbA1c, there were 6 studies (16–19, 22, 24) included in the meta-analysis. A total of 287 subjects in the control group and 321 subjects in treatment group were analyzed using random effect model. There was a statistically significant difference between patients in the control group and treatment group with a result of -0.33 (CI95%=-0.65; -0.01). The between study heterogeneity was considered low; I2 = 12% (**Figure 4**).

**Figure 5:**
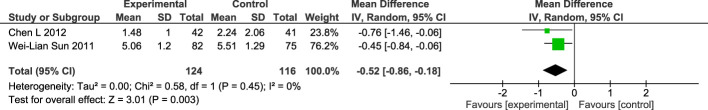
Forest plot for the change of CRP between baseline and 3-months follow-up.

There were 2 studies included in the meta-analysis of CRP changes in 3 months (21, 24). A total of 116 subjects in the control group and 124 subjects in treatment group were analyzed using random effect model. There was a statistically significant difference between patients in control group and treatment group with a result of -0.52 (CI95%=-0.86; -0.18). The between study heterogeneity was considered low; I2 = 0% (**Figure 5**).

**Figure 6:**
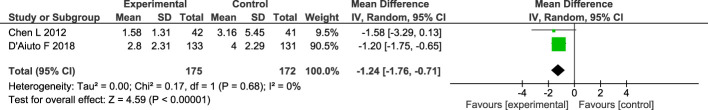
Forest plot for the change of CRP between baseline and 6-months follow-up.

As for 6 months changes in CRP, there were 2 studies included in the meta-analysis of CRP changes in 6 months (22, 24). A total of 172 subjects in the control group and 175 subjects in treatment group were analyzed using random effect model. There was a statistically significant difference between patients in control group and treatment group with a result of -1.24 (CI95%=-1.76; -0.71). The between study heterogeneity was considered low; I2 = 0% (**Figure 6**).

## Discussion

In the present systematic review and meta-analysis, it was concluded that periodontal initial treatment was beneficial for HbA1c levels and CRP of the patients with DM in the 3-months and 6-months follow-up period. When the screening of the literatures, 17 studies met the inclusion and exclusion criteria. During quality assessment process, there were 6 studies without available mean and standard deviation. 11 studies with available data were eventually included in the meta-analysis. Examining the individual studies that constitute the forest plot for the change of HbA1c at 3-months, some found that periodontal treatment significantly improved HbA1c (19, 21, 25, 26), while others did not achieve a significant difference (16–18, 20, 23, 24). This discrepancy in results was also observed for HbA1c at 6 months. However, by conducting a meta-analysis, we were able to consolidate the results of several articles and conclude that initial periodontal treatment led to a significant improvement in HbA1c. Similar to our study, the recent Cochrane report showed the effectiveness of periodontal therapy for reduction of HbA1c and CRP in type 1 and 2 diabetic patients (27). Different from the Cochrane report, our study included only type 2 diabetic patients. As for type 1 diabetes, there is no clear-cut report of HbA1c reduction by periodontal treatment, and it is classically known that elevated inflammatory markers such as CRP are related to insulin resistance (HOMA-IR) rather than insulin secretion capacity (HOIMA-β) (28). We therefore focused our study only on type 2 diabetes. We believe that this meta-analysis provided novel and clear-cut findings. We could not perform meta-analysis at the period of 12 months or longer because of lack of available data. However, a study observing changes in HbA1c and CRP at 12 months showed that improvements in HbA1c and CRP at 6 months persisted at 12 months (22), suggesting that the results of the meta-analysis at 6 months would persist at 12 months.

Removing the source of infection in the oral cavity by periodontal treatment not only improves periodontal condition and long-term prognosis of teeth, but also contributes to improvement of HbA1c without significant side effects. SRP is not a simple alternative for hypoglycemic drugs, but as a reference, we present an example of cost-effectiveness comparison as below. The cost of full-mouth SRP is about 15,000 yen (about 4,500 yen if 70% of the cost is covered by insurance) in Japan. In diabetic patients with severe periodontal disease, the improvement in HbA1c obtained with initial periodontal treatment has been reported to be generally around 0.5%. On the other hands, as to drug therapy, there are seven types of hypoglycemic drugs currently available in Japan. Among them, metformin, a biguanide (the first-line drug for diabetes treatment by the American Diabetes Association and the European Association for the Study of Diabetes) 1500 mg/day for 26 weeks has been reported to improve HbA1c by 0.56% (29). If metformin is used for 6 months, which is the most common period of reports for periodontal treatment and HbA1c, the prescription, dispensing, and drug costs would be approximately 20,214 yen (approximately 6,064 yen if 70% of the cost is covered by insurance). Thus, from a medico-economic point of view, the effect of periodontal treatment on diabetes might have significant advantages.

Periodontitis is a localized inflammatory condition that can trigger low-grade chronic inflammation. This occurs through the spread of periodontal bacteria via the bloodstream or the release of inflammatory mediators from periodontal tissues into circulation (30). As a result, it adds to the overall systemic inflammatory load, increasing the risk of various chronic diseases such as cardiovascular disease cardiovascular disease (CVD) (30), T2DM (31), kidney disease (32), cancer, and neurodegenerative disorders (33). Based on one possible mechanism mentioned above, the benefits of periodontal treatment are not limited to diabetes but are expected to spread to various inflammatory diseases in light of the principle that removal of the source of infection improves systemic inflammation. In fact, Luthra et al. (34) reviewed and concluded that treatment for periodontitis reduced CRP of various systemic inflammation disease at the 6-months timepoint. Our results, that periodontal treatment reduced both HbA1c and CRP, support the mechanism. However, since RCTs with longer follow-up periods are lacking, further studies will be required.

In this meta-analysis, intervention is integrated into periodontal treatment, but a detailed description reveals a mixture of treatments: some studies included SRP alone (16), others included SRP + OHI (18, 20, 23), others included SPR + OHI + MET (19, 26), and still others included periodontal surgery as indicated (21, 22). As for the control group, some studies included no intervention (16, 21, 23–25), while others included OHI (17–20). Particularly heterogeneous is the study by Khader et al. (17) in which full-mouth extractions were performed as intervention. This meta-analysis summarizes 10 RCTs on periodontal treatment for HbA1c reduction at 3 months, but even if the Khader paper were excluded from the analysis, the results of a significant reduction in HbA1c would still be validated (data not shown). In the future, a meta-analysis should be performed, if possible, to collect studies in which the intervention and control groups have more homogeneous conditions.

Several limitations should be noted. Firstly, pooled effects of meta-analysis could be influenced due to confounding factors, such as gender, experiences of periodontal therapy and BMI. However, there were limited studies that we could include in meta-analysis, we could not show the influence of them. Secondary, this meta-analysis only captured changes in diabetes-related items between the intervention and non-intervention groups, and did not provide detailed individualized classification of genetic or congenital responsiveness to periodontal treatment. Third, the data may be subject to error due to the mix of ITT and PPS analyses. More RCTs with a unified methodology are also needed in the future.

## Conclusion

There is robust evidence indicating that periodontal treatment reduces HbA1c and CRP levels at the 3- and 6-month timepoints. It is suggested that periodontal treatment could be beneficial rather than existing pharmacotherapy for diabetes, both in terms of side effects and in terms of medico-economic point of view. Future research should determine whether (a) the systemic effects following periodontitis treatment are sustained over time and (b) these effects hold true when assessed using a standardized methodology.
